# The roles of ABCB1/P-glycoprotein drug transporters in regulating gut microbes and inflammation: insights from animal models, old and new

**DOI:** 10.1098/rstb.2023.0074

**Published:** 2024-05-06

**Authors:** Lauren Stoeltje, Jenna K. Luc, Timothaus Haddad, Catherine S. Schrankel

**Affiliations:** Department of Biology, San Diego State University, 5500 Campanile Drive, Life Sciences North, Room 321, San Diego, CA 92182, USA

**Keywords:** ABC transporters, abcb1, P-glycoprotein, MDR1, IBD, bacterial small molecules

## Abstract

Commensal enteric bacteria have evolved systems that enable growth in the ecologic niche of the host gastrointestinal tract. Animals evolved parallel mechanisms to survive the constant exposure to bacteria and their metabolic by-products. We propose that drug transporters encompass a crucial system to managing the gut microbiome. Drug transporters are present in the apical surface of gut epithelia. They detoxify cells from small molecules and toxins (xenobiotics) in the lumen. Here, we review what is known about commensal structure in the absence of the transporter ABCB1/P-glycoprotein in mammalian models. Knockout or low-activity alleles of ABCB1 lead to dysbiosis, Crohn's disease and ulcerative colitis in mammals. However, the exact function of ABCB1 in these contexts remain unclear. We highlight emerging models—the zebrafish *Danio rerio* and sea urchin *Lytechinus pictus*—that are poised to help dissect the fundamental mechanisms of ATP-binding cassette (ABC) transporters in the tolerance of commensal and pathogenic communities in the gut. We and others hypothesize that ABCB1 plays a direct role in exporting inflammatory bacterial products from host epithelia. Interdisciplinary work in this research area will lend novel insight to the transporter-mediated pathways that impact microbiome community structure and accelerate the pathogenesis of inflammatory bowel disease when perturbed.

This article is part of the theme issue ‘Sculpting the microbiome: how host factors determine and respond to microbial colonization’.

## 1. Introduction

Cellular defence systems dictate organismal survival in an increasingly changing world. Key to survival is the ability to distinguish between microbial communities that offer metabolic or protective benefits from those of opportunistic pathogens that seek to exploit or damage host tissues. This is particularly relevant within epithelia of the gastrointestinal (GI) tract. These cells interact with trillions of bacteria and their metabolic by-products. Community composition is strongly attenuated by both bacterial and host-derived molecules and pathways [1,2].

In this context, the immune system is arguably the most heavily studied for its role in recognizing, tolerating or destroying microbial entities. The interface of the immune system with microbes is most often considered within the dichotomy of a host proteins interacting ‘with’ commensal microbial-associated motifs or ‘against’ pathogen-associated molecular patterns (MAMPs and PAMPs, respectively). However, it is also becoming increasingly clear that microbes generate an enormous number of metabolic by-products that influence many aspects of host physiology [2,3], as well as the community structure of the tolerated gut microbial communities themselves (reviewed in [4–6]). How these small molecules are regulated from a host perspective is less understood.

We seek to highlight elements of a parallel cellular protective system—the ‘chemical’ defence system—that works alongside the immune system in helping the host gut navigate beneficial and pathogenic bacteria. This protective system is composed of many types of proteins that mediate the absorption, distribution, metabolism and excretion of toxins, reactive metabolites and non-self-derived small molecules [7]. These designated ‘xenobiotic’ molecules can occur naturally from dietary and bacterial by-products, or unnaturally, from anthropogenic pollution and industrial toxins.

Although many components of this system are active in the gut [7], we will focus on transporters of the ATP-binding cassette (ABC) superfamily [8]. ABC transporter genes are present in all kingdoms of the tree of life [9–11]. The primary function of type I ABC transporters is to decrease the entry of xenobiotics into the cell. These may be pumped out either as the parent compound directly upon entry (i.e. before undergoing any biotransformation; phase 0 defence), or as metabolites following phase I or phase II biotransformation reactions (phase III transport) [7]. In the gut, known substrates include glucocorticosteroids, methotrexate, antibiotics, immunosuppressive agents and other metabolic products.

**Figure 1.**
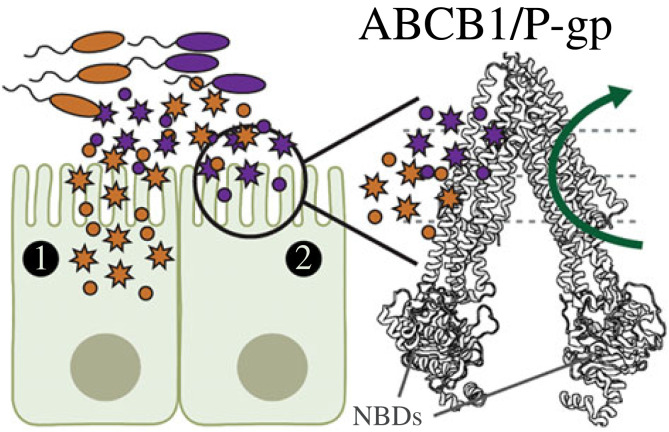
The ABC transporter ABCB1/P-gp acts as a xenobiotic gatekeeper in gut epithelia. Multiple small molecules generated by commensal or opportunistic pathogens can enter the host gut epithelia by various mechanisms ('1'; see box 1 in main text). ABCB1 protein (also known as P-glycoprotein; encoded by the *mdr1a* gene), is proposed to export (green arrow) small molecules generated from luminal bacteria ('2'). Nucleotide binding domains (NBDs) form an ATP-binding cassette region that catalyzes the efflux of interloping xenobiotics out of host epithelial cells.

ABCB1, also known as P-glycoprotein ([12]; encoded by the gene *MDR1* [13]) is one of the most heavily studied ABC transporters (figure 1). This is because the over-expression of ABCB1 in cancer cells bestows a multidrug resistance (MDR) phenotype (reviewed in Robey *et al.* [14]). More endogenous roles of ABCB1 include protection at the blood–brain barrier (BBB) and the gut epithelial sites.

Unexpectedly (or perhaps serendipitously, as often is the case in discovery-based research), the creation of the first knockout mouse model—originally generated to study ABCB1 in the BBB [15]—revealed spontaneous generation of irritable bowel disease (IBD) phenotypes in the gut [16]. However, the emergence of IBD symptoms in the *mdr1a-/-* mice required commensal colonization. Indeed, low-activity variants of ABCB1 in the human population have now been linked to a higher susceptibility to Crohn's disease (CD) and ulcerative colitis (UC). Hallmarks of these events in both species include commensal dysbiosis and epithelial inflammation. An important avenue for future work is to understand at the mechanistic level how a lack of proper ABCB1 activity impacts the maintenance of commensal communities, and in parallel accelerates the pathogenesis of gut diseases.

In this review, we will briefly discuss how microbial-derived small molecules create niches that favour gut commensal communities. We review what is known about microbial community structure in the absence of ABCB1 in humans and mammalian models. We then highlight two emerging animal models—the zebrafish *Danio rerio* and the sea urchin *Lytechinus pictus*—that are poised to help us dissect the fundamental mechanisms of ABCB1 in the direct or indirect regulation of microbial products and community structure. We hypothesize that the lack of ABCB1 activity causes xenobiotic molecules from commensals and pathogens to accumulate in host gut epithelia. Future work should identify the bacterial-derived compounds that are enriched in transporter-null animals, how their immune systems respond to this driver of inflammation, and which elements of these phenomena remain conserved in the deuterostome lineage leading to mammals.

## 2. The gut-specific bacterial small molecule world: friend or foe?

### (a) Overview of the mammalian gut niche

The GI tract encompasses the largest surface area of the body that is exposed to microbial communities (reviewed in Roda *et al.* [17]). Connecting the stomach to the bowels, the intestines begin with the caecum. Then follows the small intestines, composed of the duodenum, jejunum and the ileum. The small intestine is where most nutrient absorption occurs, from digested content moving through the intestinal lumen. The gut tract ends with the colon (or large intestine) and then the rectum.

The gut barrier is composed of the intestinal epithelium covered by an overlying mucus layer. Bacteria colonize the upper layer of the gut epithelium. This layer is composed of intestinal epithelial cells (enterocytes), which encircle the intestinal lumen. These cells have tight junctions between them, providing a physical cell barrier. Within the gut epithelium, there are goblet cells that secrete mucus to coat the apical end of the enterocytes facing the lumen, providing a second layer of protection. Paneth cells in the small intestine secrete antimicrobial peptides that also help shape the composition of the gut microbiome (reviewed in Wallaeys *et al.* [18]). The epithelial cells of the intestine can suffer from chronic inflammation if enteric dysbiosis or infection occurs. Although other important players contribute to the intestinal system, such as adaptive immune cells, dysfunction of the epithelial cells is the hallmark of IBD [19].

### (b) The microbial small molecule world within the gut niche

The significance of the small molecule profile in microbial community structure has been reviewed in depth elsewhere (see Hibbing *et al.* [4], Scherlach & Hertweck [5] and Weiland-Bräuer [6]). Briefly, the secretion of secondary metabolites and other chemical mediators are key features of microbe–microbe interactions. For example, xenobiotics and their metabolites can provide the substrates for selective growth of certain bacterial species, aid in communication or facilitate other modes of niche establishment [20].

Microbial metabolites and other products can also signal to or cross through the host gut epithelial barrier directly [3,21–23] (see box 1). Certain types of these molecules can perpetuate dysbiosis and in turn, disease, when accumulated in epithelial cells and blood or serum (reviewed in Sharon *et al.* [3]). Key examples include short-chain fatty acids (SCFAs), histamine and spermine, lipid metabolism by-products, succinate, lactate and tryptophan metabolites (more recently reviewed in Sartor & Wu [2], Kayama *et al*. [25] and Iyer & Corr [26]). However, as our knowledge on these bacterial products grows exponentially, our understanding of how host systems mediate the entry and *exit* of these compounds across the gut barrier remains comparatively stagnant.

Box 1. **Mechanisms of bacterial product entrance into epithelia.**

Growing evidence demonstrates the magnitude of bacterial metabolites that can enter blood or serum levels by various methods of translocation through gut epithelia (for a comprehensive review, see Sharon *et al.* [3] and literature cited within). The biochemical nature of different specialized microbial metabolites, from small polar molecules to larger peptides, affects their ability to cross the intestinal epithelium on their own or with the aid of other proteins. These are summarized here:

**passive diffusion:** based on biochemical properties such as size, polarity and lipophilicity, as well as degree of ionization, many metabolites and drugs can move directly through the plasma membrane, especially down large concentration gradients. This category includes many classic ABCB1 substrates [24];

**facilitated diffusion and direct import:** other hydrophilic molecules reversibly bind carriers that also travel down concentration gradients. The molecules can then cross the epithelium by using facilitated passive diffusion. In other cases, host importers, typically of the solute carrier superfamily, that are present in the apical membrane can use energy to selectively import compounds against low concentration gradients (reviewed in Sharon *et al.* [3]);

**paracellular transport (between cells):** metabolites that cannot be absorbed by transcellular transport (whether passive or facilitated) are taken up by paracellular transport;

**pinocytosis:** metabolite or small peptide uptake occurs as apical membrane protrusions engulf the fluids and any contents of the solution present outside of the host cell; and

**bacterial membrane extracellular vesicles (EVs):** more complex cargo created by bacteria can be disseminated across the cell by EVs (reviewed in Ellis *et al.* [21]). EVs contain virulence factors such as proteins (e.g. enzymes, adhesins and toxic peptides) or non-protein antigens (e.g. lipopolysaccharide) (reviewed in Sartorio *et al.* [22]).

In cases of facilitated diffusion, import, or paracellular transport, the host uses cellular machinery to actively acquire the metabolites it needs. However, this can be context dependent even for the same type of molecule. For example, SCFAs are usually transported through the epithelial barrier actively by monocarboxylate transporters (e.g. SLC5A8, SLC16A1), but can also enter cells passively by diffusion [23]. We predict that the most inflammatory substrates of ABCB1 enter the host cell through passive diffusion, or microbially-mediated paracellular transport and EVs (see main text).

## 3. Host negotiations with microbial xenobiotics, through the lens of epithelial ABC transporters

ABC transporters are ideal candidates for helping the host manage its bacterial loads (figure 1). The basic structure of an ABC transporter consists of a set of hydrophobic transmembrane domains and a hydrophilic cytoplasmic region that mediates an exportation function (also deemed ‘efflux’ or ‘transport’). All transporters have highly conserved nucleotide-binding domains (NBD; also known as an ATP binding cassette, hence ‘ABC’). The NBDs catalyse the hydrolysis of ATP. The energy released in this reaction enables the protein to actively transport substrates across the cell membrane and against large concentration gradients. Most eukaryotic ABC proteins are active exporters, including ABCB1. In the context of the gut, ABCB1 may have been initially adapted towards shaping commensal structure in addition to dealing with dietary xenobiotics.

In nature, ABCB1 substrates are known to be lipophilic, neutral or positively charged compounds that can form several hydrogen bonds with the substrate-binding pocket of the transporter (reviewed in Srikant *et al.* [24]). Therefore, we predict that many metabolically derived bacterial small molecules will fit this profile. This opens the door for discovering potential ‘biological rheostatic’ functions of ABC transporters that can fine-tune gut immune responses or otherwise influence homeostasis (box 2; see also [27–30]).

Box 2. **Do ABC transporters act as biological rheostats?**

Sets of importers evolved to bring in specific compounds made by bacteria. It is fitting then that exporting transporters likewise evolved to maintain the balance of the ‘right’ small molecules between host epithelia and the gut lumen. The correct balance of molecules remaining inside the cell has significant impact. In this context, several molecular systems can function as ‘biological rheostats' to maintain intracellular homeostasis. Rheostats are proteins or complexes that can fine-tune responses to internal or external cues, such as metabolites, to adjust the activity of gene regulatory circuits and feedback loops (reviewed in Ladurner *et al.* [27]). They can do so by binding specialized histones, chromatin-modifying enzymes and activatable transcription factors (such as hypoxic-, steroid-, lipid- or sterol-responsive factors—all of which are activated by candidate or known substrates of ABC transporters).

In the immune system, rheostats can influence host interpretation of danger signals via intracellular pattern-recognition receptors (reviewed in Sharma *et al.* [28]). These dictate (or in autoimmunity, severely miscalculates) the strength of the immune response. Surface-bound proteins in the immune system, such as programmed cell death protein 1, also have rheostatic functions, and this extends to maintaining the balance of bacterial communities in the gut [29].

Connecting these types of functions to ABCB1 is not a stretch given the literature. ABCB5 transporters are already implicated as key rheostats in a cancer signalling feedback loop [30], in which they actively secrete cytokines. In summary, the mechanistic identification of rheostatic functions of ABCB1 and other transporters in gut immunity is a promising area of study.

Notably, ABCB1 shares an evolutionary history with bacteria-specific transporters [31,32], such as the LmrA transporter of *Lactococci* [33,34]. LmrA and MDR1 even export a similar spectrum of cationic amphiphilic compounds, and this activity can be blocked by mammalian ABCB1 inhibitors (e.g. reserpine and verapamil) in both systems [34].

The structural homology present between ABCB1 and LmrA suggests that mammalian ABCB1 may have, in-part, been co-opted from ancestral bacterial proteins designed to export niche-modifying compounds. Thus, ABCB1 could enable the host to prevent the entry (or sustained build-up within the cell) of structurally similar factors from ‘modern’ bacterial colonizers and opportunistic pathogens. A handful of studies have already hinted at this possibility (table 1) and are discussed throughout the rest of the main text.

**Table 1.** Potential specific bacterial substrates of ABCB1.

organism/s	candidate substrate	evidence
mouse; human	*Listeria monocytogene*s invasion-associated proteins	*in vivo:* mdr1^–/–^ mice accumulate [35] [S] methionine labelled proteins from *Li. monocytogene*s [36]
		*in vitro:* human Caco-2 cell lines and competitive uptake experiments with the classic P-glycoprotein substrate, digoxin. Non-radioactive *Li. monocytogenes* proteins were able to preferentially compete with [^3^H]digoxin for ABCB1 binding in a concentration-dependent manner [36]
mouse	*Helicobacter bilis* cytolethal distending toxins (CDTs)	references [37,38] (predicted; no mechanism shown)
zebrafish	lipopolysaccharide (LPS)	animals treated with human ABCB1 inhibitor (cyclosporin A) had higher LPS accumulation in gut cells [39]

## 4. Impaired ABCB1 in mammals reveals a functional link with commensal tolerance and the development of irritable bowel disease

Commensal bacteria and related dysbiosis have long been hypothesized to play a central role in the development of IBD [25,35,40]. IBD is categorized by the chronic inflammation of the GI tract. UC and CD are the two main conditions under this overarching term of IBD. In both UC and CD, the mucosal lining of the intestinal lumen thins as the enterocytes thicken and inflammation increases (reviewed in Strugala *et al.* [41]). Approximately 3.1 million American adults have one or another form of IBD. There is no known single origin of this disease, nor can inflammatory bowel disease be fully cured.

### (a) ABCB1 knockout mice suffer from commensal-dependent inflammation indicative of irritable bowel disease

Mice deficient in ABCB1 (*mdr1^–/–^*) were first created in 1994 by Schinkel and collegues [15], yet Panwala *et al*. [16] were the first group to discover that *mdr1a^–/–^* mice were highly susceptible to developing severe spontaneous intestinal inflammation [16]. This mouse strain remains one of the most prominent mouse models of IBD to date. The pathology of normally colonized mice resembled human UC in several histopathological parameters: decreased phosphorylation of tight junction proteins, weight loss and decreased gut barrier function were all significant in *mdr1a^–/–^* mice [42]. The colitis-like phenotype only appears if mice were born and raised under conventional housing conditions. *Mdr1a^–/–^* mice raised germ-free or treated with antibiotics remained symptom-free. Because of this link with commensal colonization, it was hypothesized that protective ABCB1 activity was specific to the gut epithelia.

This was formally tested by generating chimeric mice. Panwala and co-authors found that irradiated *mdr1a^–/–^* mice that received wild-type (WT) bone marrow still developed colitis spontaneously, whereas irradiated WT mice that received *mdr1a^–/–^* bone marrow cells did not develop disease [16]. These findings suggest that defective *mdr1a* expression on a radiation-resistant element, probably the epithelial cells lining the gut. The results solidified that the colitis observed in *mdr1a^–/–^* mice was unlikely to be linked to normally *mdr1a*^+/+^ adaptive immune cells that respond to breaches in barrier functions (reviewed in Bossenec *et al.* [43]). Despite the absence of *mdr1a* expression on immune cells in knockout mice, Panwala *et al.* did not find significant observable defects in the overall immune system [16].

Overall, this study was the first published suggestion that ABCB1 could help mediate gut homeostasis by exporting potentially inflammatory by-products of commensals. This opened the door to speculation that expression of ABCB1 along the GI tract during development could help shape the community structure as it becomes colonized. In turn, perturbation of this function for ABCB1 may predispose animals to inflammatory disease.

### (b) ABCB1 knockout mice develop altered microbiomes

In both mice and humans, the intestinal commensal community evolves from low complexity at birth into a more diverse profile as the gut matures and transitions to increasingly complex diets [44–46]. The seminal Panwala *et al.* report identified variations in the abundance of *Proteus mirabilis, Enterococcus, Lactobacillus, Staphylococcus and Bacillus* spp*.* in *mdr1a^–/–^* mice [16]. However, the authors were unsuccessful in identifying a sole species that could be responsible for triggering inflammation [16].

A trio of follow-up studies by Dommels, Collett, Nones and colleauges [47–49] more substantially established that *mdr1a^–/–^* mice had significant alterations in their microbiota compared to WT mice. Differences in the caecal microbiota between *mdr1a^–/–^* and WT mice were observed at 12 weeks (prior to the onset of visible inflammation). This was anchored by the enrichment of *Bacteroides fragilis, Bacteroides thetaiotaomicron, Bacteroides vulgatus* and an uncultured alpha-proteobacterium, at the expense of more diverse community structure present in WT mice [49]. A reduction in bacterial diversity and total count in the *mdr1a^–/–^* mice was also found at 25 weeks, coinciding with the onset of significant intestinal inflammation. At this stage of disease progression, *Escherichia coli* and *Acinetobacter* sp. were found to be unique to the *mdr1a*^*–/−*^ mice. Similar observations of reduced bacterial numbers and species diversity within the *Bacteroidetes* and *Lachnospiraceae* subgroups have been reported in inflamed patients with IBD compared to non-inflamed individuals and healthy subjects [40,50].

### (c) ABCB1 knockout mice are impaired against pathogenic bacteria

The impact of missing ABCB1 activity extends to encounters with enteric pathogens, revealing additional consequences. For example, inoculation of *mdr1a^−/−^* mice with *Helicobacter bilis* accelerated the development of colitis [37]. *Helicobacter bilis* is known to cause inflammation in other murine IBD models. This acceleration might occur from the actions of cytolethal distending toxins [38], potentially indicating a role for ABCB1 in protecting the enterocyte against these and potentially other toxins.

Several other lines of evidence support a role of intestinal ABCB1 in limiting pathogenic invasion and dissemination (table 1). For example, both mouse and human ABCB1 proteins were shown to efflux radiolabelled *Listeria monocytogenes*' virulence proteins that are necessary for host invasion [36]. Furthermore, *mdr1a^–/–^* mice exhibited enhanced burden of *Li. monocytogene*s after infection, when compared with WT. By contrast, overexpression of ABCB1 in Caco-2 intestinal epithelial cells resulted in increased resistance to *Li. monocytogenes* infection [36]. Other bacteria, such as *Salmonella typhimurium*, actively repress ABCB1 expression at the transcriptional level in host intestinal epithelial cells [51]. Overexpression of ABCB1 *in vitro* likewise improved resistance to this enteric pathogen [52]. Although lacking in exact mechanisms, these studies demonstrate that pathogens have coevolved ways to specifically target ABCB1, suggesting that host ABCB1 proteins may directly transport bacterial peptides or toxic compounds out of the gut epithelia during pathogenic infection (table 1).

### (d) Genome-wide association studies in human populations paint a complex picture for the impact of ABCB1 alleles in human populations

The impact of human ABCB1 gene single-nucleotide polymorphisms (SNPs) with IBD susceptibility has long been studied [53]. Three most frequent variants in the *MDR1/ABCB1* gene are C3435T, G2677T/A and C1236T, which generate SNPs located in exons 26, 21 and 12, respectively. Investigations into the association between *MDR1* polymorphisms and IBD reveal complex findings, owing to the lack of standardization across collection and statistical methods.

#### (i) C3435t (exon 26)

Investigations into the C3435T polymorphism from German, Scottish and Irananian populations reported increased TT genotype frequencies associated with UC but not CD [54–56]. Meta-analyses suggest a modest association between the C3435T polymorphism and UC, but do not show a significant association with CD. Conflicting reports emerged from a study in central Poland, where C3435 allele frequencies did not significantly differ between IBD patients and controls [57].

Stronger associations were found by Ardizzone *et al.*, who identified that Italian sub-populations carrying the mutant 3435T allele had a threefold increased risk for developing CD with ileocolonic localization compared to individuals without the allele [58]. A comparable study discovered that individuals homozygous for the mutant 3435T allele had a twofold reduction in ABCB1 expression in their duodenal biopsy samples compared to individuals homozygous for the WT C3435 allele [59]. This reduction in ABCB1 expression was associated with higher digoxin plasma concentrations after oral administration, suggesting reduced efflux of the drug from the intestine owing to low intestinal ABCB1 levels. However, the researchers also noted that the 3435C→T mutation in exon 26 of the ABCB1 gene is a synonymous SNP [59]. To explain this contradiction, they proposed a potential linkage disequilibrium between SNPs in exon 26 (C3435T) and exon 21 (G2677T/A). This suggests that the observed differences in ABCB1 expression initially attributed to the exon 26 SNP might actually be the result of the associated tri-allelic polymorphism in exon 21 [60].

#### (ii) G2677t/A (exon 21)

The G2677T/A SNP leads to a non-synonymous alteration in the amino sequence from an alanine to either a serine or threonine at position 893 (Ala893Ser/Thr). The Ala893 variant exhibits significantly lower transporter activity, compared with the 893Ser variant, as measured *in vitro* and in patients [9]. Similarly, independent studies in Slovenia and the UK identified correlations between different alleles of the 2677T allele (893Ser) and the incidence of UC [61,62]. However, a comprehensive meta-analysis performed by pooling data from available studies failed to establish a significant association between allele and genotype frequencies of the G2677T/A SNP and IBD [63]. Haplotype transmission disequilibrium test analyses of Ala893Ser/Thr and C3435T suggest that the Ala893 allele, by itself, is significantly associated with the disease, while the C3435T polymorphism alone does not show a significant association [63]. This is in contrast with other work that observed no evidence for association with the 3435T polymorphism by either case-control or family-based association [54].

#### (iii) C1236t (exon 12)

In exon 12, a synonymous SNP known as C1236T has been found to exhibit linkage with the C3435T and G2677T/A SNPs [64]. This linkage suggests a higher frequency of these three SNPs occurring together on the same chromosome than what would be expected by chance alone [64]. Indeed, the 1236CT genotype and 1236T allele carriers were more frequent in IBD patients than in controls [57]. However, the associations did not reach statistical significance.

In summary, the impact of ABCB1 alleles remains clear in some studies and controversial at best in others. The discrepancies among the SNP studies performed to date can be attributed to effects from linkage disequilibrium, factors such as population heterogeneity, sample size, selection of control populations, incomplete description of disease phenotypes of UC and CD, and limited statistical power to detect moderate effect sizes.

## 5. Emerging models for studying ABCB1 in gut homeostasis and microbial management: zebrafish and echinoderms

Mouse models have provided valuable insight into the complex links between ABCB1, commensal dysbiosis and IBD pathogenesis in humans [65]. However, knockout mice are complicated by the presence of many adaptive immune cell types that would normally express ABCB1. Mice in general are less amenable to non-invasive imaging or high-throughput screening *in vivo*. Fortunately, nature has provided us with many other organisms that have these advantages to study ABCB1-specific function. We next cover two emerging model systems that we feel will be transformative for dissecting the fundamental mechanisms of ABCB1 in gut commensal homeostasis and pathogenic defence: zebrafish and sea urchins, with a focus on transgenic sea urchins. In the *in vitro* realm, three-dimensional organoids and similar culturing techniques also provide parallel systems in which to test ABCB1 direct functions against luminal bacteria (box 3) [66–70].

Box 3. **Organoid systems for studying host–microbe interactions.**

While the primary focus of this review is to highlight emerging animal models for studying the specific roles of ABCB1 at the intersection of gut luminal contents and host inflammation, several *in vitro* advances merit brief description. Most notably, **intestinal-derived organoids** (IO; also called **enteroids**) are self-organizing three-dimensional cell cultures that can contain multiple differentiated cells derived from gut-specific stem cells [66]. As such, they exhibit functional and architectural similarities to real intestines. This includes several physiological properties such as barrier function, mucus production, and selective absorption (reviewed in Bartfeld *et al.* [67] and Ahn *et al.* [68]). Typical IOs have a ‘basal-out’ construction, that is, the apical side of epithelial cells face inward, as in the natural gut lumen. Recent advances have enabled polarity switches such that IOs can also be grown ‘apical-side out’. Another approach is to grow **organoid-derived monolayers** (ODMs). These are established by dissociating three-dimensional grown IOs and seeding them onto a Transwell plate. In ODMs, the apical surface is exposed directly to media. Although the monolayers cannot be maintained for long periods of time via passaging, this culture method enables easier access to the apical side of cells via the culturing media. It also provides more convenient sample collection compared to classic IOs.

In the context of studying ABCB1 protein function in gut epithelia, *MDR1*-knockout human enteroids and ODMs were recently developed [69]. The overall morphology, barrier integrity, gene expression changes and growth of the organoids were not affected by knockout of the *MDR1* gene. These tests were done in the absence of bacterial colonization, however. Co-culture of IOs or ODMs with specific bacteria can provide a high-throughput platform for mechanistic study of host–microbe interactions. For traditional IOs, microinjection of bacteria directly into the IO lumen is needed for bacteria to have contact with the apical side of the epithelium. Although initially a labour-intensive process, organoid microinjections can now be done on high-throughput platforms. For ODMs and ‘inside-out’ IOs, bacteria can be added directly to the media. Expanding these organoid models to understand the flow of microbial-derived small molecules in and out of host cells under different conditions (e.g. *MDR1* knockout) would provide a powerful study system.

We propose that by exposing *mdr1*-knockout mouse, rat and human-derived organoids to luminal bacteria, small molecule profiles can be analysed more closely in the absence of ABCB1 transporter activity. The use of organoid models also promises great potential for personalized treatment for patients (reviewed in Poletti *et al.* [70]) that have IBD-relevant single nucleotide polymorphisms in the *MDR1* gene. Patient-derived two- and three-dimensional organoids can likewise be subject to microinjection of varying microbiota, allowing researchers to conduct more high throughput analysis of how their own ABCB1 variants function against specific communities of bacteria.

### (a) Zebrafish

The zebrafish *D. rerio* is a well-established model for development, ecotoxicology, and more recently, IBD [71,72]. The ABCB1/MDR1 homologues *abcb1a* and *abcb1b* were originally mis-identified in the genome [73]. Follow-up synteny analysis clarified that a paralogue *abcb4* instead exists, and that its protein product closely phenocopies mammalian ABCB1 in several aspects [74]. First characterized in depth by the Luckenbach group and colleagues [75,76], zebrafish *abcb4* is localized ubiquitously in the embryo and becomes enriched in the larval intestine. Morpholino knock-down of *abcb4* increased accumulation of human ABCB1-specific fluorescent substrates and toxic compounds within embryos.

Robey *et al.* [77] further characterized abcb4 functions in the BBB and Lu *et al*. [78] identified that highest abcb4 messenger RNA expression exists in the intestine, followed by the liver, and then muscle, gill and other organs. In a study to test microbial substrates of abcb4, zebrafish larvae were exposed to lipopolysaccharides (LPS, a component of the outer membrane of Gram-negative bacteria) in the presence and absence of the ABCB1 inhibitor cyclosporine A [39]. Higher LPS accumulation in the GI region was found in the individuals treated with cyclosporine A, suggesting that LPS could be a direct substrate of abcb4 in fishes ([39]; table 1).

More recent CRISPR/Cas9-based experiments have generated the first homozygous mutant of abcb4 in zebrafish [79]. Although this study focused on the effects of knockout in the BBB, mutant embryos exhibited a higher accumulation of known fluorescent substrates in the GI tract. Importantly, the adaptive immune system in zebrafish only matures after three weeks [80], therefore the new *abcb4*-null line will be informative for studying base epithelial function before abcb4^+^ lymphocytes enter the fold. This model organism is now well situated to model epithelial-specific ABCB function in the context of the microbiome.

### (b) Marine invertebrates: worms and echinoderms

Studies of invertebrates have contributed greatly to fundamental processes involved in human health and disease [81]. A conserved role for ABCB1 in helping hosts negotiate with bacterial products is evident deep within the tree of life, particularly in marine environments. For example, embryonic and adult stages of the echiurid worm, *Urechis caupo*, develop in mudflats that contain metabolic products of bacteria and to some extent, plants: these animals naturally exhibit levels of ABCB1-like efflux activity that are higher than many MDR-positive tumours [82,83].

**Figure 2.**
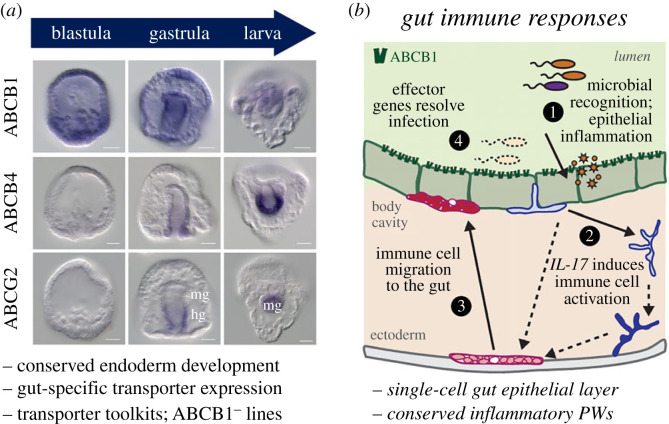
A simple echinoderm model for testing the regulation and function of ABC transporters in host-microbe interactions. (*a*) The sea urchin develops a feeding larva with a tripartite gut by 48 hours (*Lytechinus pictus*) to 72 hours (*Strongylocentrotus purpuratus*) post-fertilization. Millions of embryos are generated in a single spawn. ABC transporters are placed in specific sections the developing gut tube (mg, midgut; hg, hindgut) and concentrate in the larval gut. Scale bars: 50 μm. Modified from Schrankel & Hamdoun 2021. (*b*) An organismal-wide response is easily imaged in optically clear larvae. Gut epithelial responses are facilitated by epithelial recognition of microbes. In the case of dysbiosis or infection, epithelial cytokine signaling activates immune cell migration and effector gene responses. Additional advantages of the sea urchin system are listed. PWs, pathways.

The pelagic zone of marine ecosystems is also rich with bacteria and their secondary metabolites. Echinoderms, such as sea urchins, must navigate this zone for several months as feeding larvae before they initiate metamorphosis into the adult form [84]. Species such as *Strongylocentrotus purpurpatus* and *Lytechinus pictus* spawn and develop externally and have been excellent model organisms for development [85–88], xenobiotic metabolism [11,83,89,90] and innate immunity ([91]; figure 2).

Here, we argue this model can be adapted for the study of ABCB1 for several reasons. First, following fertilization, the sea urchin embryo develops a gut that contains three distinct functional compartments [92] by 48–72 hours post-fertilization, depending on the species. Sea urchins share an evolutionary history with vertebrates (the deuterostome lineage); thus their endodermal specification and differentiation pathways are deeply conserved at the molecular level [88]. ABC transporters have been mapped in early sea urchin development and revealed remarkable compartmentalization in the nascent gut tube, including homologous of ABCB1 ([93,94]; figure 2*a*). Second, animals are optically clear during early development, making them amenable to imaging. Indeed, fluorescent substrate and inhibitor assays [95] in living embryos illuminated that ABCB-specific protein activity is active in the gut during gastrulation and feeding [94].

Third, pan-16S ribosomal RNA probes indicate that larval guts are colonized following initial feeding on microalgae [96]. When larvae undergo dysbiosis or are exposed to opportunistic pathogens, such as *Vibrio diazotrophicus*, a stereotypical immune response occurs (figure 2*b*): enterocytes thicken significantly, immunocytes called pigment cells migrate to the inflamed gut, and a coordinated molecular response anchored by the cytokine IL-17 occurs across the animal [97,98]. Notably, transcript levels of the sea urchin *ABCB1* homologue increase in *Vibrio-*exposed larvae [98]. Bacterial exposure assays have been adapted to model several aspects of host–commensal interactions in larvae [96]. Collectively, these studies suggest that the sea urchin ABCB1 may share fundamental aspects of transporter function in gut microbial defences.

## 6. Conserved gut inflammatory phenotypes are prominent in ABCB1-knockdown sea urchin larvae

**Figure 3.**
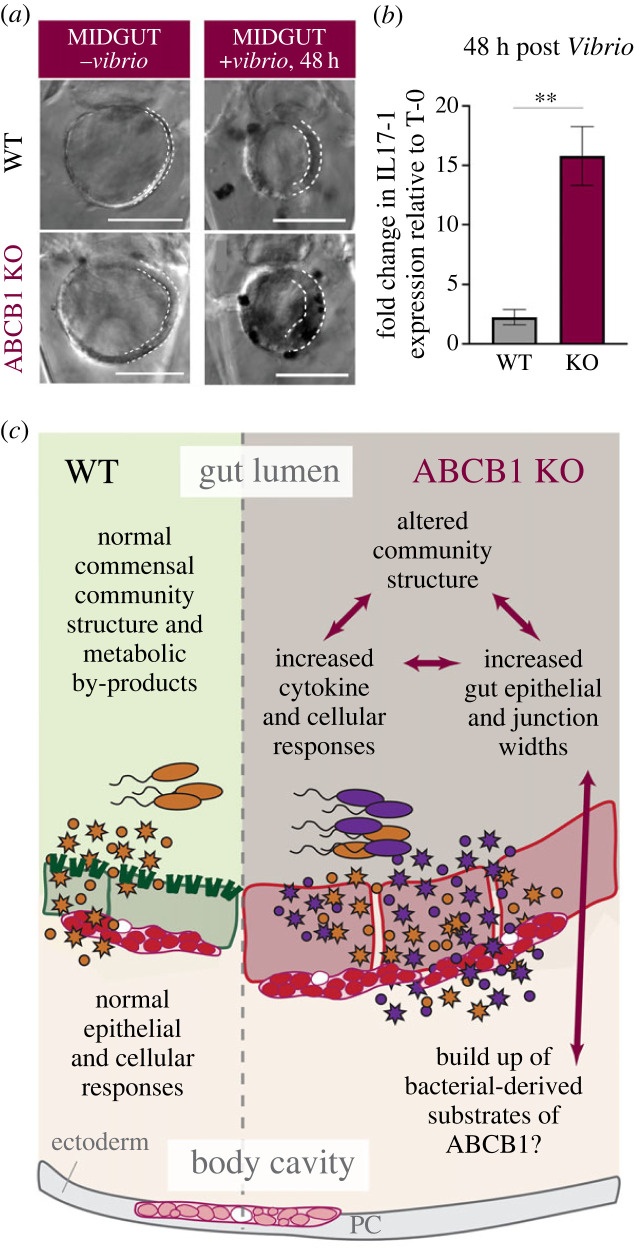
Inflammatory phenotypes suggest a buildup of bacterial metabolites in CRISPR/Cas9-mediated ABCB1 knockout in the sea urchin. (*a*) Magnified view of larval sea urchin guts in cross section. ABCB1 knockouts (KO) exhibit thickened epithelia (outlined in dotted white line) and gut-localized pigment cells prior to exposure to *V. diazotrophicus*. These phenotypes were exacerbated upon exposure to the pathogenic *Vibrio* species. Scale bars: 50 μm. Modified from Fleming *et al.* 2021. (*b*) IL-17 cytokines remain activated 48 hours post bacterial exposure in ABCB1 KOs, whereas wild type (WT) animals have resolved a spike in IL-17 expression. Modified from Fleming *et al.* 2021. (*c*) Schematic for hypothesized interactions between ABCB1 and luminal bacteria. KO, knockout; PC, pigment cell (larval immunocyte); WT, wild type. These phenotypes extend to other animal models (see main text).

To test the impact of ABCB1 on gut homeostasis, the Hamdoun and Schrankel laboratory groups targeted the *S. purpuratus ABCB1* locus by CRISPR/Cas9 [99]. We found that *SpABCB1*-knockdown larvae (also called ‘crispants’) showed no obvious morphological differences compared to WT, but exhibited an increased accumulation of fluorescent substrates in the gut [99]. Morphological differences in the gut epithelia became evident once feeding began (figure 3*a*). Larval midgut epithelia showed significant inflammation and increased targeting by migratory immune cells, hallmarks of the colitis phenotypes observed in the original *mdr1a^–/–^* mouse model of IBD [16]. We next looked at the possible function of SpABCB1 to protect larvae from pathogenic microbes. We exposed crispants to *V. diazotrophicus* and assessed gut morphology, pigment cell (immunocyte) behaviour and cytokine responses. Gut inflammation and pigment cell migration to the gut were significantly increased in SpABCB1 mutants, mirroring the phenotypes of *mdr1a^–/–^* mice further exposed to different pathogens ([37]; figure 3*a*).

We also found that ABCB1 crispants infected with *Vibrio* exhibited higher levels of *Sp-IL-17-1* and *Sp-IL-17-4* cytokine activation compared with *Vibrio*-infected control larvae (figure 3*b*). In mice, humans and sea urchins, microbial activation of the cytokine IL-17 in gut epithelia will control the recruitment of immune cells to the gut and helps repair tissue damage from inflammation in an autocrine manner [98,100,101]. In mammals, IL-17C becomes dysregulated in dextran sulfate sodium-induced colitis models [100]. At the time of this publication, epithelial IL-17 responses have yet to be detailed in the *mdr1a^–/–^* mouse or *abcb4^–/–^* zebrafish, but this is an exciting area for future research in these animal models.

In summary, our results in the sea urchin support an ancestral role for ABC transporters in host–microbe interactions and, to our knowledge, we are the first to suggest that in the absence of ABCB1, gut epithelial IL-17 signalling can be activated at greater levels and for longer periods of time following bacterial infection. We hypothesize that initial gut inflammation may be indicative of a failure to resolve commensal-derived xenobiotics in this animal (figure 2*c*). A buildup of microbial factors may subsequently drive the trio of stronger inflammatory responses we have observed. We also suspect that Sp-ABCB1 transporter activity aids in the elimination of specific *Vibrio*-derived compounds. Previous studies showed that *V. diazotrophicus* will translocate through the gut into the body cavity [97], signifying the potential utilization of virulence factors. These may include known zonula occludens toxins [102,103], and/or small diffusible molecules required for quorum sensing, niche establishment or virulence. Additional pathogenicity factors such as secreted extracellular proteases and toxins could be generated through type-II secretion systems prominent in *Vibrio* species [104].

### (a) *Lytechinus pictus* is a genetically enabled sea urchin species for future work on ABC transporters in host–microbe interactions

One caveat of our initial ABCB1 perturbation study in sea urchins was that the CRISPR/Cas9 mutations were generated and assessed only in the F_0_ generation, which is inherently mosaic for mutations until breeding is done to create true homozygous mutants. This study was limited by the life cycle of *S. purpuratus*, whose generation time can take up to 2 years.

To address this, our research groups have recently adopted the species *Ly. pictus* owing to its advantageous characteristics, including a short generation time (less than three months) [105] and the recent availability of its genome sequence [106]. Capitalizing on these characteristics, we successfully generated a knockout mutant line for the *Ly. pictus* homologue of *ABCB1* [90]. We designed a dual guide RNA approach targeting the first NBD, which is crucial for efflux function. Through comprehensive sequencing analysis and substrate accumulation assays, a successful 800 bp deletion was confirmed in the germline of F_0_ generation. Subsequently, the mature heterozygous mutants were subjected to in-crossing, leading to the production of homozygous mutant F_2_ larvae [90].

These animals are now mature adults bred to the fourth generation. Pilot studies have identified gut epithelial differences between WTs and mutants during gastrulation and in feeding larval stages, as well as altered community profiles of gut bacteria (L.S. and C.S.S., 2023, unpublished observations). Future work will dissect the impacts of ABCB1 at the molecular and mechanistic level in this model.

## 7. Conclusion and future directions

**Figure 4.**
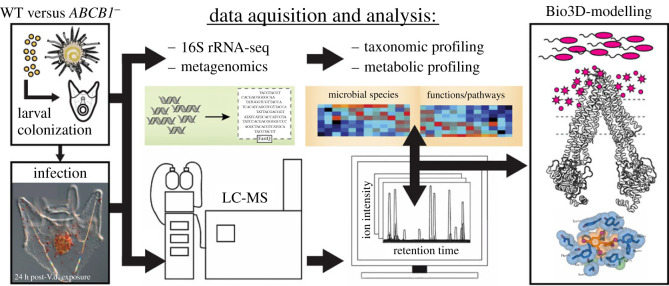
Future directions for identifying the bacterial-derived compounds that are ABCB1 substrates. Metagenomic and metabolomic profiling, combined with the advent of increasingly powerful 3D-modeling and prediction approaches, can be used in the sea urchin system (pictured on the left), given the extreme fecundity of animals created per spawn. These techniques can be applied to other model organisms or human patients and datasets to identify areas of conservation and differences in ABCB1 function against bacterial small molecules. LC-MS, liquid chromatography-mass spectrometry.

Xenobiotic defence systems are fundamental to organismal survival at the cellular level. There is perhaps no site more relevant for this function than the gut. Here, ABCB1 and other transporters are exquisitely positioned to limit the absorption of substances that the cell perceives as harmful. It is evident that reciprocal interactions between bacteria and host ABCB1 proteins in the gut have coevolved. The current research paradigm of ABCB1 in the intestinal tract must be expanded upon to include the regulation of the small molecule environment shared by commensals. Animals that share fundamental processes in gut development and structure may provide clues as to how transporter activity (or lack thereof) directly contributes to gut homeostasis or disease, respectively. A fusion of metabolomics, metagenomics, and three-dimensional-modelling approaches (figure 4) in a wide range of organisms will help us address several outstanding questions:

(i) **are there common bacterial metabolites and virulence factors favoured for efflux by ABCB1 or other efflux transporters?** Systematic metabolic and metagenomic screens are needed to link metabolites with host systems that manage microbial friends and foes [1,3]. These can be assisted with powerful three-dimensional-Bio [107,108] and predictive protein modelling (e.g. AlphaFold [109]) of known transporter structures with candidate substrates. Follow-up biochemical and bacterial mutagenesis studies can be used to validate these interactions at the transporter protein level;
(ii) **has the preference of these compounds remained conserved across evolutionary time?** Sampling from diverse taxa will help us resolve whether small molecule substrates from different types of bacteria share substrate affinity for ABCB1. Alternatively, we may find that different species co-opted or adapted transporter sub-families to negotiate with gut bacteria more specific to their environments;
(iii) **how do transporters get allocated across sections of the gut tract as it is being built and colonized?** Crosstalk between host and commensals relies on the systems developed and deployed in gut epithelia. These are controlled by conserved developmental pathways, which can be studied and verified *in vivo* in model organisms such as the sea urchin or zebrafish;
(iv) **how do transporter polymorphisms dictate epithelial detoxification efficiencies in the context of luminal bacteria?** As protein prediction modelling accelerates at a breakneck pace, we may be able to soon predict the allelic substrate preferences of ABCB1 proteins against candidate small molecules. Complementary *in vitro* and *in vivo* studies are needed to clarify mechanistically how ABCB1 defects may influence host and microbial xenobiotic metabolism and subsequent commensal compositions; and
(v) **how does transporter activity dovetail with other modes of microbial regulation and crosstalk with host systems?** The other papers in this issue address some fascinating concepts and new paradigms for host control of its microbiome. It will be of great interest to understand how ABCB1 activity works in concert with these systems to attenuate the small molecule profile in the gut lumen.

## Data Availability

This article has no additional data.
